# Diffuse Large B-Cell Lymphoma in Kenya: MYC, BCL2, and the Cell of Origin

**DOI:** 10.1200/JGO.18.00203

**Published:** 2019-05-02

**Authors:** Jonathan Wawire, Shahin Sayed, Zahir Moloo, Aliyah R. Sohani

**Affiliations:** ^1^ Aga Khan University, Nairobi, Kenya; ^2^ Massachusetts General Hospital, Boston, MA; ^3^ Harvard Medical School, Boston, MA

## Abstract

**PURPOSE:**

Diffuse large B-cell lymphoma (DLBCL) is the most commonly diagnosed non-Hodgkin lymphoma in adults in Kenya. Cell of origin (COO) and double expression of MYC and BCL2 are two important prognostic factors for DLBCL. A small subset (5% to 10%) of DLBCL cases show positivity for CD5 and are associated with poor prognosis, whereas CD30 antigen, seen in up to 10% of cases, may be a useful target for therapy. We sought to determine the prevalence of MYC/BCL2 double expression, COO, and proportion of Epstein-Barr virus positivity among patients with DLBCL diagnosed at a tertiary referral laboratory in Kenya.

**PATIENTS AND METHODS:**

All cases of DLBCL diagnosed from 2012 through 2015 in our pathology department were analyzed. Tumor tissue microarray sections were stained with CD20, CD3, CD5, CD30, BCL2, BCL6, CD10, MUM1, MYC, and Ki67, classified for COO on the basis of the Hans algorithm, and subjected to Epstein-Barr virus-encoded small RNAs in situ hybridization.

**RESULTS:**

Among 165 DLBCL cases, the median age was 50 years, and there was no sex predilection. Only 18 (10.9%) cases showed double expression for MYC and BCL2. Germinal center B (GCB)-cell type DLBCL accounted for 67 cases (40.6%) and 97 cases (59.4%) were classified as non-GCB. The mean Ki67 proliferation index was significantly higher in the double-expressing (45%) and non-GCB groups (36%) compared with the non–double-expressing group (29%) and GCB group (26%). Sixteen cases (9.7%) were Epstein-Barr virus-encoded small RNAs positive, 12 (75%) of which were non-GCB.

**CONCLUSION:**

DLBCL in Kenya is seen in much younger patients with the poor prognostic non–GCB-type accounting for 59.4% of cases. MYC and BCL2 double expression was seen in fewer tumors than reported in the literature and in significantly older patients.

**CONTEXT**

**Key Objective**
To determine the prevalence of MYC/BCL2 double expression, cell of origin (COO), and Epstein-Barr virus status of diffuse large B-cell lymphoma (DLBCL) cases diagnosed at a tertiary referral laboratory in Kenya. The study highlights the clinicopathologic characteristics of DLBCL from Kenya using immunohistochemical staining panels.
**Knowledge Generated**
This study highlights key differences between DLBCL cases diagnosed in Kenya and cases diagnosed in Western countries, including a younger median age (50 years) at presentation, a higher proportion of non-germinal center B COO DLBCL (59.4%), and a lower percentage of double-expressing cases (9.7%).
**Relevance**
The high proportion of poor prognostic non-germinal center B-cell type group from Kenya underscores the need for routine testing of patients with DLBCL for COO to identify those patients who would benefit from addition of rituximab to their treatment.

## INTRODUCTION

Diffuse large B-cell lymphoma (DLBCL) is the most commonly diagnosed non-Hodgkin lymphoma in adults.^1^ It is a heterogeneous disease with varying clinical outcomes attributable to its biology and molecular pathogenesis. Important prognostic factors for DLBCL are revised international prognostic index,^2^ cell of origin (COO),^3,4^ presence of MYC and BCL2 rearrangements by fluorescent in situ hybridization or standard cytogenetics,^5^ absolute lymphocyte and monocyte count, and imaging with positron emission tomography.^2,3,6^

Alizadeh et al^3^ described three molecular subgroups by gene-expression profiling (GEP) on the basis of the COO: germinal center B-cell type (GCB), activated B-cell (ABC) type, and the unclassifiable type. The GCB type DLBCL is characterized by genetic mutations in *BCL2*, *BCL6*, and *MYC* genes, with epigenetic modifications in *EZH2* genes.^7^ Patients with this subtype of DLBCL have a better prognosis compared with patients with ABC type when treated with cyclophosphamide, doxorubicin, vincristine, and prednisolone (CHOP), the standard therapy, still, for a majority of Kenyan patients with DLBCL.^8^ Addition of rituximab results in a remarkable improvement in 5-year overall survival rates (from 60% to 90%) in the GCB group.^9^ The ABC-type DLBCL is characterized by constitutive activation of nuclear factor κ-light-chain enhancer of activated B-cells (NF-κB) pathway, a protein complex that controls the transcription of DNA promoting cell proliferation.^7^ Receiving CHOP treatment alone, this subgroup of patients does poorly; the 5-year overall survival rate is approximately 35%,^3^ and only modestly improved to 44% with addition of rituximab.^9^ The unclassifiable group of DLBCL has no distinct genetic pattern and has a similar prognosis to the ABC type.^3,7,9^ It is important to determine COO in patients with DLBCL who may benefit from newer targeted therapeutic agents. The agents under investigation include ibrutinib, lenalidomide, and bortezomib, which target the NF-κB pathway in non-GCB DLBCL.^10-13^ The current World Health Organization classification requires the identification of GCB and ABC/non-GCB subtypes and incorporation of the subclassification into clinical practice.^14^

Although GEP is not available in routine practice, robust immunohistochemistry (IHC) surrogates have been developed for determining COO. The Hans algorithm uses three antibodies in sequence: CD10, BCL6, and MUM1.^15^ Tumors with greater than 30% positivity in CD10 are classified as GCB, whereas CD10-negative cases are stained additionally with BCL6 and MUM1. CD10-negative tumors that are positive for BCL6 and negative for MUM1 (cutoff of 30% staining) are classified as GCB, whereas any other combination is considered non-GCB. This algorithm is the most widely applied and has concordance rates of 80% to 87% with GEP.^16^ When applied to patients receiving CHOP alone, the algorithm has demonstrated prognostic significance between the GCB and non-GCB groups.^15^ This prognostic distinction diminishes when applied to patients receiving rituximab with CHOP (R-CHOP).^15-19^ Most patients in Kenya, as a result of limited resources, receive CHOP alone as standard chemotherapy^8,20^; hence, the use of this IHC algorithm retains important prognostic relevance in our setting.

Fluorescent in situ hybridization is used to identify specific gene rearrangements involving *BCL2* and *MYC*. Concurrent presence of *BCL2* and *MYC* rearrangements is seen in 6% to 10% of DLBCL.^5,6^ Such so-called double-hit lymphomas have a considerably poorer prognosis, with median survival rates ranging from only 6 to 13 months.^1,2,6,21^ IHC for MYC and BCL2 overexpression has been used in DLBCL prognostication, using cutoffs of 40% nuclear positivity for MYC and 50% cytoplasmic positivity for BCL2.^5,22^ Cases positive for both markers are termed double expressing and are seen at a much higher frequency (20% to 30%) than double-hit lymphomas. Cases positive for both markers have a poor prognosis that is intermediate between DLBCL not otherwise specified and double-hit lymphoma, with 5-year overall survival rates of 10% to 36%.^2,5,22-24^

Epstein-Barr virus (EBV)-positive DLBCL has increasingly been reported in immunocompetent patients younger than 50 years.^25^ Data suggest a varied morphologic spectrum with better prognostic outlook for EBV-positive DLBCL than previously described.^14^

COO and MYC/BCL2 double expression are two crucial prognostic factors in DLBCL that are recommended in the routine evaluation and reporting of this lymphoma.^14,26^ Therefore, our aim in this study was to determine the prevalence of MYC/BCL2 double expression among cases of DLBCL diagnosed at Aga Khan University Hospital, Nairobi (AKUHN), and to classify cases of DLBCL by COO. To our knowledge, this is the largest East African study to date describing critical clinicopathologic characteristics of DLBCL.

## METHODS

Formalin-fixed, paraffin-embedded tissue blocks of consecutive cases of histologically confirmed DLBCL from January 1, 2012, to December 31, 2015, were retrieved from archives in the Pathology Department of AKUHN. Data on age, sex, and tumor site were abstracted from the pathology database. Hematoxylin and eosin–stained slides were reviewed with an appropriate block selected for tissue microarray (TMA) construction. Three tumor areas were circled and included in a TMA master block, which also included control cases of Burkitt lymphoma, plasmablastic lymphoma, reactive tonsillar tissue, and normal epidermis.

IHC with antibodies (namely, CD3, CD5, CD10, CD20, CD30, MUM1, BCL6, BCL2, MYC, and Ki67) was conducted on 5-micron TMA sections. The details of the antibodies used are listed in Table 1. IHC was performed on Dako EnVision FLEX Autostainer (Agilent Technologies, Glostrup, Denmark) according to the manufacturer’s specifications.

**TABLE 1:**
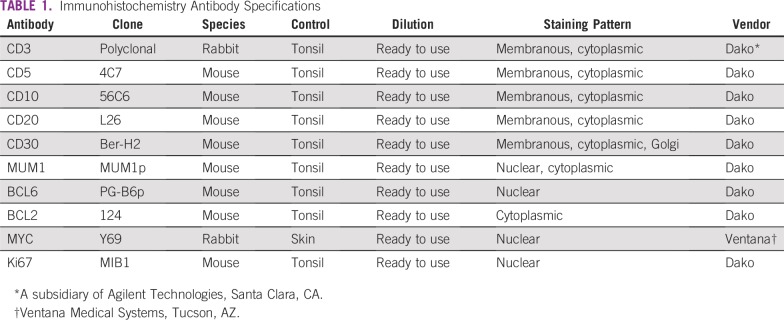
Immunohistochemistry Antibody Specifications

Scoring, in 10% increments, was done by counting 100 tumor cells at a magnification of ×400. Cutoffs for positivity were applied as follows: 30% membrane staining for CD20, CD10, CD5, and BCL6^15^; 50% cytoplasmic positivity for BCL2^5,22^; 30% and 40% nuclear positivity for MUM1 and MYC positivity, respectively^5,22^; and 20% membrane positivity for CD30.^27^

Per the Hans algorithm,^15^ cases in the GCB category were as follows: CD10-positive cases, CD10-negative but BCL6-positive cases, and MUM1-negative cases. Non-GCB patients included all those negative for both CD10 and BCL6 or that were CD10 negative but MUM1 positive. Cases were considered double-expressing DLBCL when both BCL2 and MYC were positive.^5,22^ In addition, all cases were subjected to Epstein-Barr virus-encoded small RNAs (EBER) in situ hybridization,^28^ which was performed using a Leica BOND-III automated immunostainer with a Leica Bond Ready-to-Use ISH EBER Probe according to manufacturer’s instructions (Leica Biosystems, Buffalo Grove, IL).

We conducted statistical analysis with SPSS, version 23 (IBM, Armonk, NY) and included descriptions of median age, sex preponderance, tumor site (whether nodal or extranodal), and proportions according to COO and MYC/BCL2 status. The *t* test was used to calculate the level of significance in the median age and mean Ki67 for the different groups; Fisher’s exact test was used to calculate the level of significance for correlation between COO, tumor site, and double expression.

## RESULTS

**FIG 1:**
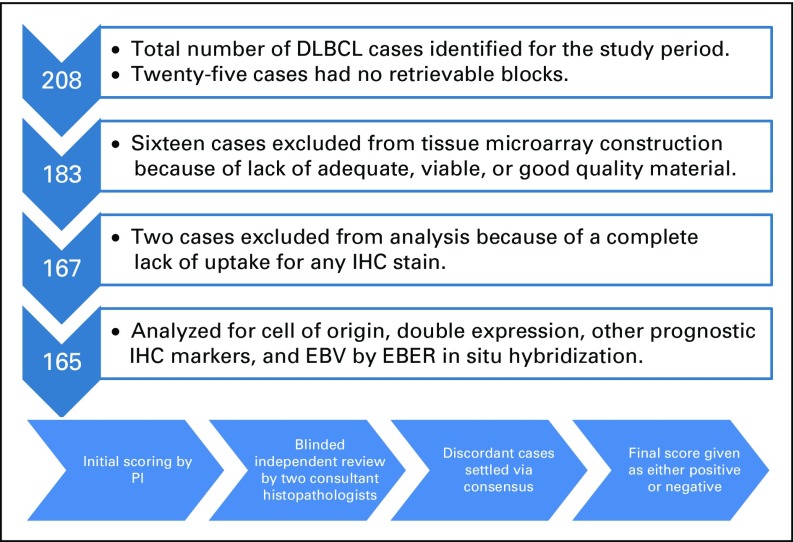
Flowchart illustrating selection of cases. From 208 cases identified in the pathology database, blocks were available for 183 cases, of which 16 were excluded for lack of quality material, leaving 167 cases to be included in the tissue microarray. Two cases did not take up any immunostain. The final total of cases analyzed for MYC/BCL2 double expression and cell of origin was 165. DLBCL, diffuse large B-cell lymphoma; EBER, Epstein-Barr virus-encoded small RNAs; EBV, Epstein-Barr virus; IHC, immunohistochemistry; PI, principal investigator.

A total of 208 cases were identified for the study period. Blocks were retrieved for 183 of the cases, from which 16 were excluded for lack of adequate viable tissue and were not included in the construction of the TMA blocks. Two TMA blocks were constructed with a total of 167 cases and 20 controls. After TMA construction, another two cases were excluded from additional analysis, because of failure of uptake of any immunostain. A flowchart for the selection and inclusion of cases and their subsequent evaluation is listed in Figure 1.

### Clinical Characteristics

There were 90 men (54.5%) men and 75 women (45.5%); the median age at diagnosis was 50 years (range, 20 to 90 years). A total of 95 cases (57.9%) were from nodal sites, and 69 (42.1%) were extranodal. The mean Ki67 proliferation index was 30% (range, 10% to 100%). These results are listed in Table 2.

**TABLE 2:**
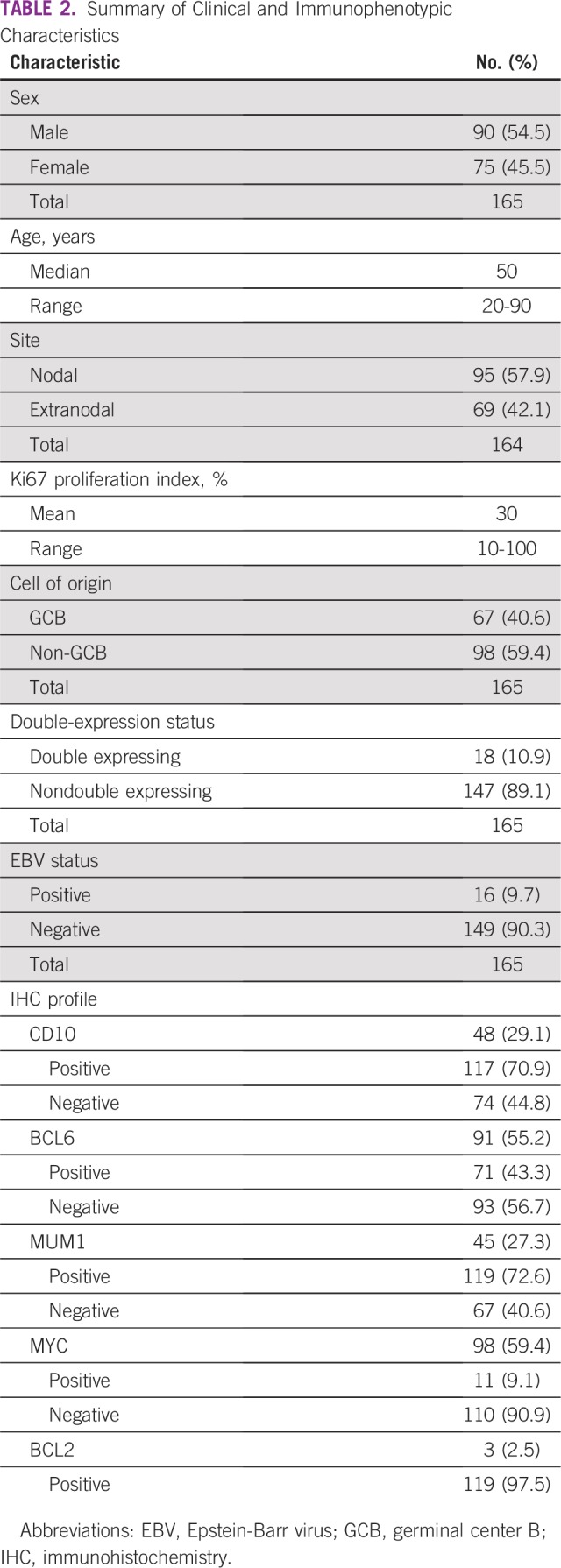
Summary of Clinical and Immunophenotypic Characteristics

### Double Expression of MYC and BCL2

Only 18 patients (10.9%) had double expression of both MYC and BCL2, and the median age of these patients (61 years) was significantly higher compared with that of the non–double-expressing group (49 years; *P* = .0178).

The mean Ki67 proliferation index for the double-expressing group was significantly higher than that of the non–double-expressing group (45% *v* 29%, respectively; *P* = .0309), with no association between double-expression status and nodal versus extranodal tissue site, sex, or COO (Table 3).

**TABLE 3:**
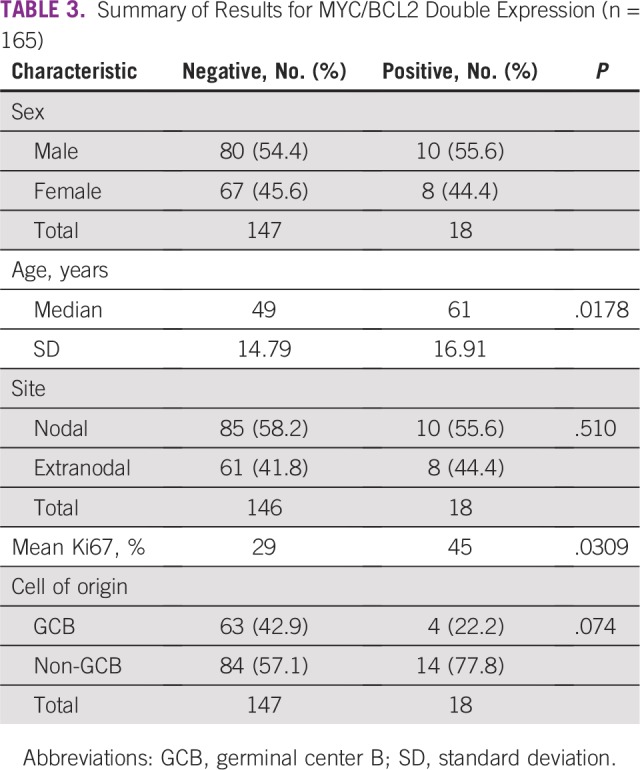
Summary of Results for MYC/BCL2 Double Expression (n = 165)

### Cell of Origin

All 165 cases were analyzed for COO using the Hans algorithm. CD10 and BCL6 were positive in 29.1% and 44.8% of cases, respectively, and MUM1 was positive in 43.3% of cases. Overall, 67 cases (40.6%) were GCB-type DLCBL and 98 cases (59.4%) were non–GCB-type DLCBL.

There was an association between COO and tumor site, with a greater likelihood of non-GCB cases being nodal than extranodal (*P* = .016). The mean level of Ki67 expression was higher in the GCB group (36%) compared with the non-GCB group (26%; *P* = .0088), with no significant difference in age between the GCB and non-GCB groups (Table 4).

**TABLE 4:**
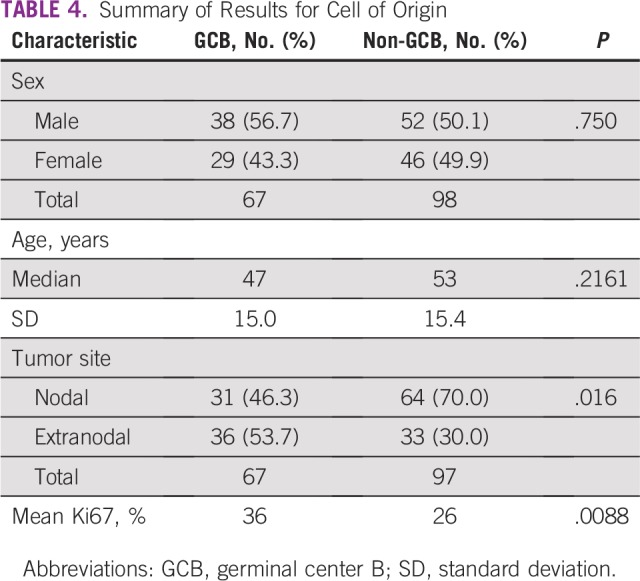
Summary of Results for Cell of Origin

### CD5 Expression

All CD5-positive cases stained negative for cyclin D1, excluding the diagnosis of mantle cell lymphoma. Additional comparison with CD3 staining was made to exclude possible reactive T cells among the tumor cells. Eleven of 121 cases (9.1%) stained positive for CD5, 10 occurring in men, with no significant differences in age, mean Ki67 expression, tumor site, or COO between CD5-positive and negative cases.

### CD30 Expression

Only three of 119 cases (2.5%) were positive for the CD30 antibody. This low number precluded additional subgroup analysis.

### EBV Status

Sixteen cases (9.7%) were positive for EBV by EBER in situ hybridization. Twelve of these cases (75%) were non-GCB and 12 of the positive cases were seen in nodal sites (10 of them non-GCB; Table 5).

**TABLE 5:**
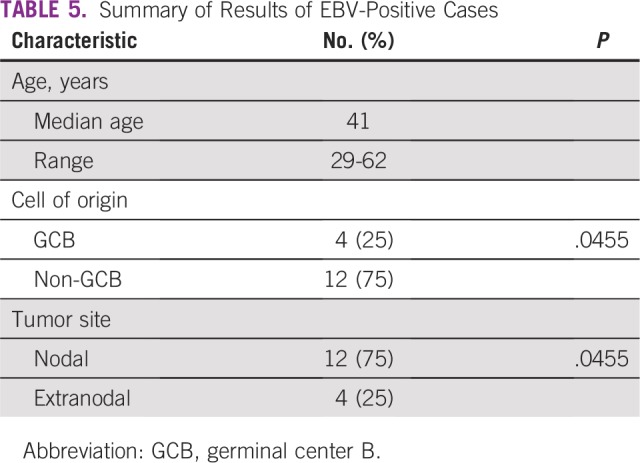
Summary of Results of EBV-Positive Cases

## DISCUSSION

To our knowledge, this is the largest study conducted of the immunophenotypic characteristics of DLBCL in Kenya evaluating the prevalence of double expression and COO, as well as the proportion of CD5- and CD30-positive cases. We also used MYC antibody in evaluation of DLBCL cases in Kenya.

The median age of 50 years in our study is similar to that reported by Naresh et al^8^ and 10 years younger than in Western populations.^1,29,30^ In their survey of 95 cases in sub-Saharan Africa, Naresh et al^8^ showed a slight male preponderance (male-to-female ratio of 1.5:1). In an unpublished study, Sherman et al assessed 51 cases of DLBCL at AKUHN and found no sex predilection but highlighted that up to 37% of the cases were diagnosed in younger patients age 30 to 40 years (O. Sherman, personal communication, December 2011). Because only 6% of Kenyans are older than 55 years, it is reasonable to conclude that as our population ages, the incidence of DLBCL is likely to increase.

We identified 10.9% cases as double expressing for both MYC and BCL2 proteins. This proportion is lower than reported in other studies, which reported ranges from 20% to 30%.^1,5,22,30^ Our double-expressing cases showed a significantly higher Ki67 proliferation index, in keeping with the tumor biology, a finding replicated in the same studies.^1,5,22,30^ Double-expressing DLBCL also occurs in slightly older individuals,^2^ which we also found in the current study, with a median age of 61 years for patients with double-expressing DLBCL, compared with 49 years for patients with non–double-expressing DLBCL (*P* = .0178). The older age for double-expressing tumors may also point to why there was such a low prevalence of this DLBCL type in our study, the population of which comprised substantially younger patients. Unlike previous studies,^5,22^ no association was demonstrated between double expression and COO or site of biopsy. This may be because this study showed a lower prevalence of double-expressing tumors and therefore was not powered to demonstrate these differences.

We found 40.6% of cases to be GCB-type DLBCL, compared with other studies that showed the GCB group to account for 42% to 54% of DLBCL.^17^ There were relatively lower percentages of tumors expressing BCL6 (44.8%) and CD10 (29.1%), compared with prior studies in which BCL6 was expressed in approximately 60% of tumors and approximately 40% of tumors were positive for CD10.^1,30^ Similar studies conducted in Japan and China reported GCB ranges of 32% to 39%, suggesting a geographic variation in prevalence of this subclass of DLBCL.^31^ This study’s cases also had a higher Ki67 proliferation index in the GCB group compared with the non-GCB group (36% *v* 26%, respectively; *P* = .0088). There is conflicting evidence on the impact of a high proliferation index on prognosis, because the use of chemotherapy has been postulated to be more effective in rapidly dividing tumors.^30^ In studies by Hans et al,^15^ Choi et al,^17^ and Visco et al,^32^ GEP was used as a gold standard upon which concordance was calculated. Our study did not have a comparison with GEP. Various studies conducted using Hans algorithm have failed to replicate its prognostic utility in patients undergoing R-CHOP therapy.^18^ Nevertheless, as a result of the prohibitive cost of rituximab in our setting, most patients with DLBCL still receive CHOP as the standard therapy. Under these circumstances, the Hans algorithm as applied here still bears important prognostic utility in this setting.

Previous studies have been inconsistent in their reporting of the prevalence of MYC overexpression. A study by Hu et al^24^ showed a MYC overexpression of 64% (n = 468), whereas Horn et al^33^ reported a prevalence of 31.8% (n = 282). Johnson et al^22^ demonstrated MYC expression in 29% of their study cases (n = 167). In the current study, we showed MYC expression in 27.3% of the cases analyzed, a proportion comparable to that reported by Johnson et al^22^ and Green et al.^5^ The MYC-positive tumors had higher mean Ki67 expression compared with MYC-negative cases (43% *v* 25%, respectively; *P* < .001). This is an expected finding, because tumors with MYC overexpression are more aggressive and have a higher proliferative capacity.^34^ This study showed no preponderance for extranodal sites among MYC-positive cases, contrary to what has been demonstrated in other studies.^33,34^ Results are inconsistent regarding the prognostic value of MYC protein overexpression alone. Ho and Rodig^34^ reported poor overall survival for patients positive for MYC overexpression, whereas Johnson et al^22^ and Green et al^5^ suggest that MYC overexpression alone does not portend a worse prognosis unless present in combination with BCL2 overexpression, as seen in double-expressing lymphomas.

BCL2 protein was overexpressed in 40.6% of the cases in the current study compared with 50% reported in studies elsewhere.^22,24,33^ In a local unpublished study, the percentage of BCL2-positive cases was even lower, at 18% (n = 51; O. Sherman, personal communication, December 2011). BCL2-positive cases in our study were predominantly non-GCB (61.2%) and seen at a higher frequency in nodal sites (61.2%). These findings did not reach statistical significance but were consistent with the literature.^4,17^ BCL2 overexpression does not carry any prognostic significance on its own, especially in the rituximab era.^5^

CD5-positive cases accounted for 9.1% of cases in our study, a similar proportion to the 5% to 10% reported in the literature,^35^ with 10 of the 11 cases occurring in men. More cases were seen in extranodal sites, consistent with reported literature,^36^ although too few cases were seen in our study to reach statistical significance. The same was seen with COO, for which most CD5-positive cases were non-GCB, a finding consistent with the literature.^1^ This subgroup of patients has poorer outcomes, with a 5-year overall survival of 34% compared with 50% in CD5-negative DLBCL. These patients also have higher rates of CNS recurrence.^35^ It is likely that CD5 expression confers resistance to chemotherapeutic agents by altering the tumor microenvironment and reducing apoptosis.^35,36^ Additional clinical information should be sought in such cases regarding the patient’s HIV status, because CD5-positive tumors tend to occur at higher frequency in patients with HIV.^1,35^

CD30, positive in Reed-Sternberg cells of classic Hodgkin lymphoma, is also expressed in anaplastic large-cell lymphoma and a subset of DLBCLs. In one study of 903 patients with DLBCL, 14% were positive for CD30 and were associated with improved 5-year overall survival regardless of COO.^27^ The CD30-positive group also showed unique molecular signatures associated with downregulation of NF-κB, which provides a plausible genetic basis for its superior outcomes, in addition to being amenable to CD30-directed monoclonal antibody therapy with brentuximab vedotin.^27^ In our study, CD30-positive cases accounted for only 2.5% of the cases studied, a proportion much lower than that reported in other studies (10% to 20%).^19^ Additional studies are recommended to better characterize the value of routine staining for CD30 antibody in DLBCL in Kenya.

EBV-positive DLBCL, thought to account for 3% to 15% of cases of DLBCL, was reported in older (> 50 years) immunocompetent patients and was associated with poorer outcomes than EBV-negative DLBCL.^37^ However, recent data suggest a wider age range of EBV-positive DLBCL, with better outcomes.^25^ In our study, approximately 10% of patients were EBV positive and ranged in age from 29 to 62 years (median age, 41 years). As reported in other studies, most cases occurred in nodal sites and were of the non-GCB subtype.^38^ Data on the immune status and long-term outcome of these patients were not available for this study and present an opportunity for future studies.

In summary, we report that DLBCL in Kenya occurs in younger patients (median age, 50 years). Most patients present with nodal disease; there seems to be no predilection for either sex. Although Hans algorithm used to classify COO for DLBCL is of limited utility in prognosticating patients receiving R-CHOP treatment, we still find it relevant in our setting, where most patients receive CHOP as standard treatment for DLBCL. Of note, 59.4% of our study patients had the unfavorable non–GCB-cell type of DLCBL, highlighting a large group of patients who need rituximab added to their treatment. In addition, these patients may also be considered in the future for targeted therapies such as bortezomib and lenalidomide. Double expression for MYC and BCL2 was seen in only 10.9% of patients. These patients were substantially older, which may explain in part the reason for such a low prevalence. Given the poor prognosis of MYC/BCL2 double expression, we recommend routine testing for these two markers at diagnosis despite its lower prevalence in our setting.
